# Effect of Multi-Component on Crack Resistance of High-Performance Concrete on Subway Underground Station Floor

**DOI:** 10.3390/ma15175868

**Published:** 2022-08-25

**Authors:** Shaoyun Xu, Peiwei Gao, Lingling Huang, Lifeng Chen, Feng Cen, Zhiqing Zhao, Yilang Tian

**Affiliations:** 1 Department of Civil and Airport Engineering, College of Civil Aviation, Nanjing University of Aeronautics and Astronautics, Nanjing 211106, China; 2 College of Architectural Engineering, Yangzhou Polytechnic Institute, Yangzhou 225127, China; 3 Jiangsu Sinoroad Engineering Research Institute Co., Ltd., Nanjing 211800, China

**Keywords:** underground station floor, HPC, orthogonal experiment, compressive strength, crack resistance

## Abstract

In view of the easy cracking of the high-performance concrete (HPC) of the subway underground station floor, the effects of fly ash, basalt fiber, expansive agent, and water reducer on the compressive strength, initial crack time, through-crack time, and crack area of the HPC on a subway underground station floor at different ages by orthogonal experiment are examined. Scanning electron microscopy (SEM), X-ray diffraction (XRD), and mercury intrusion porosimetry (MIP) are used to further analyze the microstructure and product composition of the optimal ratio HPC and reference concrete. The results show that with the increase in the content of fly ash and expander, the 7 d and 28 d compressive strength of the HPC gradually decreased. However, as the content of basalt fiber increased, the 7 d and 28 d compressive strength of the HPC gradually increased. The 7 d and 28 d compressive strength of the HPC increased and then decreased with the increase in water-reducer content. When the content of fly ash, basalt fiber and expander increased, the initial crack and through-crack time of the HPC delayed gradually, and the crack area gradually decreased. When the fly-ash content reached 30%, the cracking area accounted for 65.1% of the concrete with 15% fly-ash content. When the basalt fiber content reached 0.4%, the cracking area accounted for 56.5% of the concrete with 0.1% basalt fiber content. When the expander content reached 10%, the cracking area accounted for 60.5% of the concrete with 4% expander content. With the increase in the content of water reducer, the initial crack and through-crack time of the HPC gradually advanced, and the crack area gradually increased. When the water-reducer content reached 1.3%, the cracking area accounted for 105.7% of the concrete with 1.0% water-reducer content. The addition of fly ash and expander can produce a large number of crystalline products to fill the pores, and the disordered distribution of the added basalt fibers increases the compactness of the structure; moreover, the internal micro-pores increase, and the macro-pores decrease, thus improving the crack resistance.

## 1. Introduction

With the rapid development of urbanization, underground space has been developed and utilized on a large scale; as one of the most effective ways to make full use of underground space to relieve the pressure of surface traffic, the subway has developed rapidly recently. At the same time, it has brought some problems: the concrete of the subway underground station faces various constraints and loads, early cracks are hard to avoid [1], and cracking and leakage have caused the failure of underground concrete structure, resulting in huge engineering losses. The traditional anti-cracking method for concrete is a single mineral admixture or admixture [2,3,4,5,6], and mostly ordinary concrete, while the concrete of the underground subway station is mostly high-performance concrete (HPC). Therefore, evaluating the crack resistance of the HPC in subway underground stations is a huge challenge faced by engineers [7,8].

Scholars around the world have conducted a large number of experiments on the crack resistance of concrete. The shrinkage cracking performance of concrete can be improved by adding auxiliary cementitious materials [9]. Salah A. [10,11] found that wet curing of self-compacting concrete mixed with 50% and 70% slag powder for 7 days can reduce the risk of cracking. The cracking risk of 35% fly ash and 35% slag powder is much lower than that of 70 % slag powder alone, but it requires wet-curing for 3–7 d. At the same time, some studies have shown that adding fly ash can reduce the temperature increase, but it will weaken the early strength, which is not conducive to the crack resistance of concrete from the perspective of resistance [12,13]. Meddah [14] found that when silica fume is added to the concrete with water binder ratio of 0.6, the result shows that the dry shrinkage rate increases with the increase in silica fume content. Perfilov V.A. et al. [15] studied the strength and crack resistance of basalt fiber concrete and determined the relevant parameters. Ming [16] found that when steel fiber and CaCO_3_ whisker are mixed into the cement matrix, they can cooperate with each other when damaged, inhibit the generation and expansion of micro cracks and cracks, and improve the strength and toughness of the cement matrix. The mixing effect of steel fiber and polyester fiber is better, and the total crack area is reduced by 99%. Calcium and magnesium expansion agent can expand in the early and middle stages of concrete, inhibit the concrete shrinkage deformation, and the expansion amount, is easy to control, which has a good compensation effect and an anti-cracking function [17,18,19]. Hyeonggil Choi [20] simulated the volume change of concrete mixed with calcium magnesium expansive agent and established the volume change model of expansive concrete based on the water pore size distribution and water balance state. The MgO expander expands during setting and hardening, which can compensate for shrinkage and reduce the generation of cracks [21]. If the amount is too large, it will lead to safety hazards. If the amount is appropriate, the stability is good [22,23]. Babak Safaei [24] studied the effect of calcium carbonate on the strength of cement paste, and the addition of 2% calcium carbonate NPs improved the compressive strength of the samples by 25%. It is found that polycarboxylate superplasticizer will accelerate the shrinkage of concrete in some environments in the project, and its early anti-cracking ability is poor [25,26]; however, due to its advantages of high water reduction rate, simple synthesis, and good slump resistance, which is widely used in concrete engineering [27], so it is necessary to research its influence on concrete cracking under the influence of multiple components. Al-amoudi [28] found that different kinds of water-reducing agents have different effects on the plastic shrinkage strain of concrete. Ma [29] found that naphthalene and polycarboxylate superplasticizers can prolong the initial cracking time of mortar and reduce the cracking sensitivity of mortar; the inhibition effect of polycarboxylate on cracking sensitivity is better than naphthalene, and can reduce the maximum crack width of mortar. Naphthalene water-reducing agents increase the development rate of the maximum crack width of mortar. From the above research, it is found that at present, there are few studies on the effect of mineral admixture, expander, basalt fiber and water reducer on the crack resistance of underground HPC.

Scholars worldwide have used different methods to conduct concrete-crack-resistance experiments. Reza Hanifeh [30] derived two sets of the matrix/platelet displacement solutions (far-field and transient) based on the theory of elasticity. The platelet/matrix components satisfy the equilibrium and compatibility conditions and satisfy the equilibrium requirements and the overall boundary conditions. Sahmani S. [31] developed a non-classical panel model based upon the Gurtin-Murdoch elasticity theory, in conjunction with the first-order shear deformation shell theory, which efficiently considers the effects of surface-free energy. The size-dependent critical buckling loads and associated postbuckling equilibrium paths are obtained corresponding to various geometrical parameters and thermal environments. He Z. et al. [32] obtained the early performance parameters related to the crack resistance of concrete through uniaxial restraint test, which provides a basis for optimizing the crack resistance of concrete from the perspective of mix proportion. Slam [33], Altoubat [34], and other scholars studied the plastic shrinkage cracking of fiber-reinforced concrete by the plate test. The plate test is also the standard test method recommended by the current specification to evaluate the early age plastic shrinkage cracking of plain concrete and fiber-reinforced concrete [35]. Soroushian et al. [36] studied the effect of fiber on the crack resistance and reinforcement of concrete by the plate test and found that fiber has an obvious improvement effect on the plastic shrinkage and dry shrinkage cracking of concrete.

The multi-component studied in this paper is mainly mixed with different amounts of mineral admixtures, fibers, and additives. HPC for the subway underground station floor was prepared by orthogonal tests, and orthogonal experiments were designed to evaluate the influences of mineral admixture, expansion agent, basalt fiber, and water reducer on the compressive strength, initial crack time, through-crack time, and crack area of the HPC at different ages. In addition, the microstructures and product compositions of the HPC under the optimal and benchmark proportions were further studied by scanning electron microscopy (SEM), X-ray diffraction (XRD), and mercury intrusion porosimetry (MIP).

## 2. Experiment Materials and Methods

### 2.1. Materials

According to the requirements of Chinese standards, Cement: P·II 52.5 Portland cement, class I of fly ash, silica fume, slag, natural river sand with a fineness modulus of 2.56, basalt gravel with a particle size of 5–30 mm, basalt fiber, calcium oxide expander, and water reducer are used to prepare the experimental specimens. Among them, the performance indicators of cement meet the technical requirements of GB175-2007, the performance indicators of fly ash meet the requirements of GB/T1596-2005, the performance indicators of silica fume meet the requirements of GB/T 27690-2011, the performance indicators of slag powder meet the requirements of GB/T18046-2000, and the water reduction rate of the water reducer is 30–40%. The expander and water reducer meet the requirements of GB50119-2013, the chemical composition of calcium oxide expansion agent is shown in Table 1, and the performance index of basalt fiber is shown in Table 2. The basalt is short-cut basalt fiber produced in Shanghai, which meets the requirements of GB/T38111-2019.

### 2.2. Specimens Preparation and Testing Programs

In order to study the effect of fly-ash content, basalt fiber content, expansion agent content, and water reducing agent content on the crack resistance of the HPC, combined with raw material chemical composition and performance parameters, the orthogonal test of L_16_ (4^4^) is adopted. Table 3 lists orthogonal test factor level, and Table 4 lists HPC mix ratio.

**Figure 1:**
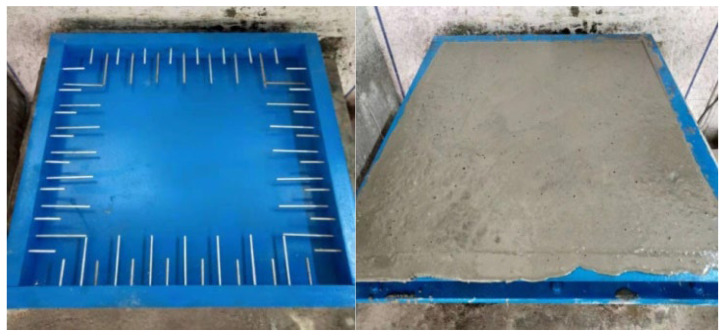
The mold and specimen.

The demould cubic specimens of 100 mm × 100 mm × 100 mm were placed in the curing chamber for curing for 7 and 28 days. According to the relevant test methods in GB-T50081-2011, the compressive strength of the cubic specimens cured for 7 and 28 days was measured with a 500 t micro-electro-hydraulic servo universal testing machine. The concrete crack resistance test was carried out according to CCES 01-2004, and the specimen of 60 cm × 60 cm × 6.3 cm was formed. The mold and specimen are shown in Figure 1.

The formed specimen was cured in an environment with relative humidity of 60 ± 5% and temperature of 20 ± 2 ℃. After the concrete was poured and leveled, the time was t_0_; when the first crack was observed, it was recorded as t_1_. After the crack appeard, it was observed and measured every 20 min until the crack penetrated; at this time, it was recorded as t_2_. It was observed and measured every hour after the crack penetrated, once every two hours after two hours, once after 10 hours, and once after 24 hours. The contents of observation and measurement include the number of cracks, the average crack width of each crack, the average crack length of each crack, the maximum crack length, and the maximum crack width.

The initial cracking time, crack penetration time, and cracking area were calculated in accordance with Equations (1)–(3):

*T*_1_ = *t*_1_ − *t*_0_ (1)

*T*_2_ = *t*_2_ − *t*_0_ (2)

(3) $$A=\sum_{i}^{N}W_{i}L_{i}$$

where *T*_1_ is initial crack time of concrete (min), *T*_2_ is crack penetration time of concrete (min), A is cracking area of concrete (mm^2^), *t*_0_ is the initial moment after concrete leveling, *t*_1_ is the momen of the first crack in concrete, *t*_2_ is the moment of concrete crack penetration, N is the total number of concrete cracks, *W_i_* is average width of the NO.i crack in concrete (mm), and *L_i_* is average length of the NO.i crack in concrete (mm).

The 10 mm × 10 mm × 5 mm samples that had been cured for 28 days were soaked in anhydrous ethanol for 3 d, and the samples were taken out and dried in an oven at 60 °C for 48 h. The JSM-6510 high-resolution scanning electron microscope was used for SEM scanning analysis.

The samples were taken from absolute ethanol that had been cured for 28 days, dried in the oven at 60 °C for 24 h, partially ground to powder, and sieved through 80 μm sieve. D8 advance X-ray diffractometer produced by Bruker Corporation (Karlsruhe, BW, Germany) was used for XRD analysis under a scanning range of 10° to 80° at a scanning rate of 0.30 s/step, step size = 0.02°. Some samples were analyzed by mercury intrusion, and the test was performed by Auto Pore IV 9510 mercury injection meter produced by the Micromeritics Instrument Corporation (Norcross, GA, USA).

**Figure 2:**
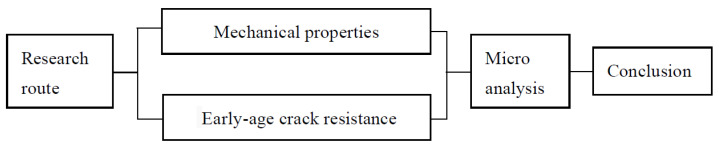
Roadmap of research.

Figure 2 shows the research roadmap of the paper.

## 3. Results and Discussion

### 3.1. Effect of Material Composition on Compressive Strength of Concrete

**Figure 3:**
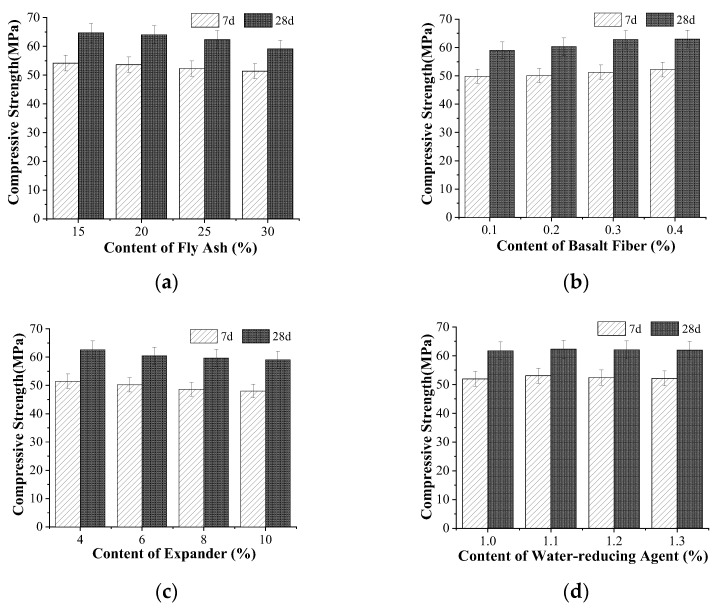
Effect of different components on compressive strength: (**a**) fly ash, (**b**) basalt fiber, (**c**) expander, and (**d**) water reducer.

The effect of each component on the compressive strength is displayed in Figure 3. Figure 3a shows that as the content of fly ash increased, the compressive strength cured for 7 and 28 days decreased gradually. When the content of fly ash was 20%, 25%, and 30%, respectively, the compressive strength cured for 7 days accounted for 98.9%, 96.5%, and 98.8% of the concrete with 15% fly-ash content, and the compressive strength cured for 28 days accounted for 98.9%, 96.3%, and 91.3% of the concrete with 15% fly-ash content, respectively. As the content of fly ash increases, the amount of cement decreases, and the Ca(OH)_2_ required for hydration of fly ash may be insufficient. At the same time, the moisture in the concrete migrates to the surface early, and the free water decreases, which affects the hydration of fly ash, so the compressive strength gradually decreases [37].

As illustrated in Figure 3b, with the increase in the content of basalt fiber, the compressive strength cured for 7 and 28 days increased gradually. When the content of basalt fiber was 0.2%, 0.3%, and 0.4%, respectively, the compressive strength cured for 7 days accounted for 100.5%, 102.8%, and 104.8% of the concrete with 0.1% basalt fiber content, and the compressive strength cured for 28 days accounted for 102.3%, 106.5%, and 106.8% of the concrete strength with 0.1% basalt fiber content, respectively. As the content of basalt fiber exceeded 0.3%, the increase in compressive strength cured for 28 days was not obvious. The three-dimensional chaotic network system formed together with the aggregate bore the external load by adding basalt fiber into the concrete, which inhibits the generation and expansion of internal cracks in concrete, limits the transverse tensile deformation of concrete specimens, and thus improves the compressive strength [38].

Figure 3c shows that as the content of the expander increased, the compressive strength cured for 7 and 28 days decreased gradually. When the content of the expander was 6%, 8%, and 10%, respectively, the compressive strength cured for 7 days accounted for 97.6%, 94.5%, and 93.3% of the concrete with 4% expander content, and the compressive strength cured for 28 days accounted for 96.7%, 95.4%, and 94.4% of the concrete with 4% expander content, respectively. Due to the slow hydration rate of CaO, CaO is still hydrated after cement hydration, solidification, and hardening, and the hydration products cause damage to the formed concrete structure, resulting in the reduction in compressive strength. With the gradual increase in the content of the expender, the expansion of calcium hydroxide formed by the reaction of CaO increases continuously, and the interface structure of the concrete is further damaged, resulting in a gradual decrease in the compressive strength.

As shown in Figure 3d, with the increase in the content of water reducer, the compressive strength cured for 7 and 28 days increased first and then decreased. When the content of water reducer was 1.1%, 1.2%, and 1.3%, respectively, the compressive strength cured for 7 days accounted for 102.1%, 100.9%, and 100.4% of the concrete with 1.0% basalt fiber content, and the compressive strength cured for 28 days accounted for 100.8%, 100.5%, and 100.3% of the concrete with 1.0% basalt fiber content, respectively. On the whole, the effect of water reducer on compressive strength was not obvious. Concrete workability is improved by adding water reducer into the concrete under the condition of a low water-cement ratio, which reduces the internal pores of the cement stone, making the structure more compact [39] and improving the strength, but the content of the water reducer needs to be reasonable. When the content of water reducer is too large, the compressive strength will be reduced.

### 3.2. Effect of Material Composition on the Time of Initial Crack and Through Crack of Concrete

**Figure 4:**
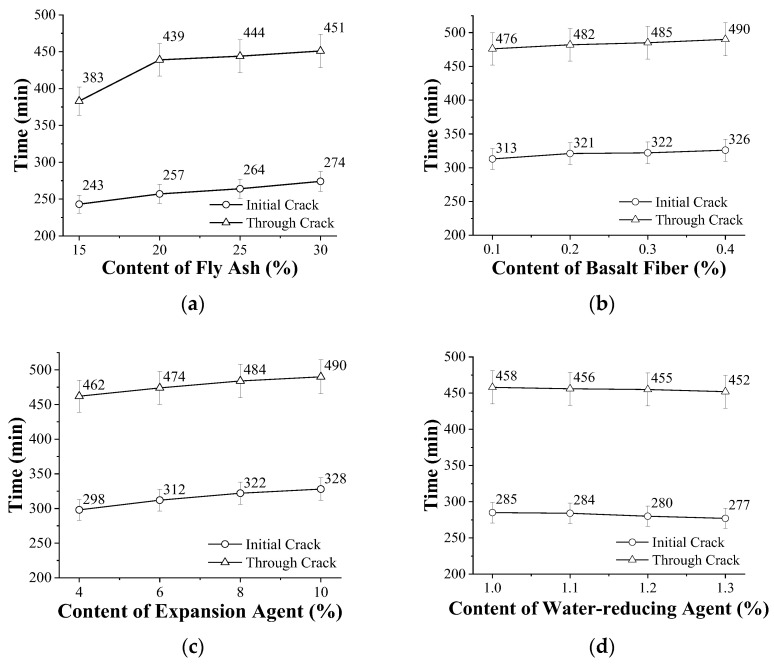
Effect of material composition on the time of initial crack and through-crack of concrete: (**a**) fly ash, (**b**) basalt fiber, (**c**) expander, and (**d**) water reducer.

Figure 4 shows the effect of each component on the time of initial cracking and through-crack of concrete. As seen in Figure 4a, as the content of fly ash increased, the time of initial crack and through-crack were gradually prolonged. When the fly-ash content reached 30%, the initial crack time was prolonged by 31 min, and the through-crack time was prolonged by 68 min, compared with the fly-ash content of 15%. As the content of fly ash increases, the proportion of cement decreases, the water demand for early hydration decreases, and there are fewer hydration products. The internal pores cannot be effectively filled, there are free bleeding channels, and the excess free water is migrated back to the surface to supplement the water loss caused by the evaporation of water to form a self-curing layer, which is beneficial to the delayed cracking of concrete and the delayed penetration of cracks, which is conducive to delayed cracking and the delayed through-crack of concrete [40].

As shown in Figure 4b, with the increase in the content of basalt fiber, the time of initial crack and through-crack were gradually prolonged. When the content of basalt fiber was 0.4%, the initial crack time was prolonged by 13 min, and the through-crack time was prolonged by 24 min, compared with the basalt fiber content of 0.1%. The addition of basalt fibers makes the interior of the concrete more compact, which can prevent the further migration of water to the surface and evaporation, reducing the plastic shrinkage, and the random distribution of basalt fibers can also resist the early autogenous shrinkage of concrete. Therefore, with the increase in the basalt fiber content, the initial crack time and through-crack time of concrete gradually increased.

As illustrated in Figure 4c, as the content of expander increased, the time of initial crack and through-crack were gradually prolonged. When the expander content reached 10%, the initial crack time was prolonged by 30 min, and the through-crack time was prolonged by 28 min, compared with the expander content of 4%. The addition of expander in concrete produces a large amount of crystalline products when it encounters water, which can fill the capillary channels and pores inside the concrete, and the addition of expansion agent can make the concrete expand moderately. In the interior of the slab, the expansion of the concrete will be transformed into the surrounding pressure, thus partially offsetting the shrinkage of the concrete. As a result, the addition of the expander delays the initial crack time and the through-crack time.

Figure 4d shows that as the content of water reducer increased, the initial crack time and through-crack time of concrete gradually shortened. When the water-reducer content reached 1.3%, the initial crack time was shortened by 8 min, and the through-crack time was shortened by 6 min, compared with the water-reducer content of 1.0%. The finer effect of capillary pores in concrete is more obvious due to the increase in the content of water reducer. As a result, the capillary pressure will increase, resulting in an increase in the self-shrinkage of the concrete, which will cause the concrete to crack and penetrate through the cracks earlier.

### 3.3. Effect of Material Composition on Cracking Area of Concrete

**Figure 5:**
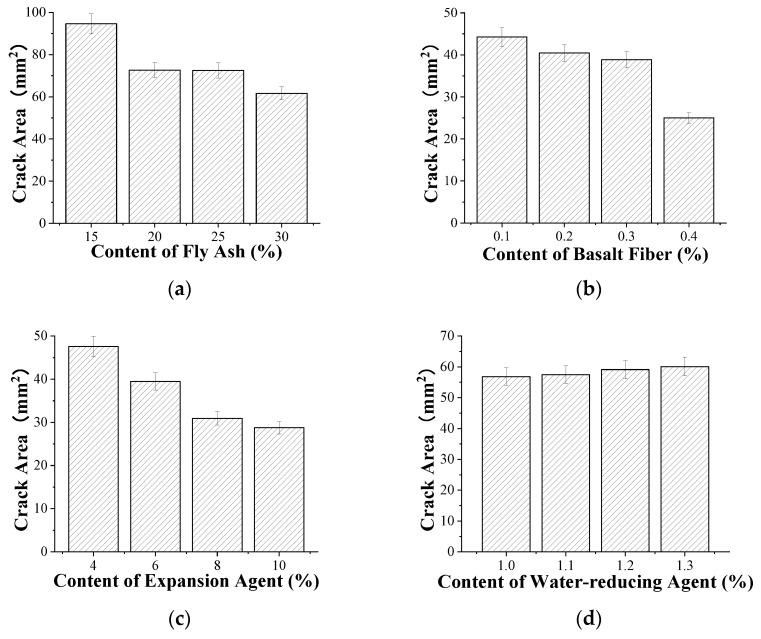
Effect of material composition on cracking area of concrete: (**a**) fly ash, (**b**) basalt fiber, (**c**) expander, and (**d**) water reducer.

Figure 5 shows the effect of each component on the cracking area of concrete. From Figure 5a, as the content of fly ash increased, the cracking area of the concrete gradually decreased. When the fly-ash content reached 30%, the cracking area accounted for 65.1% of the concrete with 15% fly-ash content. As the content of fly ash increases, the proportion of cement becomes smaller. Fly ash hardly participates in hydration at the early age, and the water demand for cement hydration decreases; therefore, the self-shrinkage is small, and the low hydration heat also reduces the temperature shrinkage caused by the difference between inside and outside temperatures. Therefore, with the increase in fly-ash content, the shrinkage deformation of concrete will gradually reduce, the cracking area of concrete will gradually reduce, and the early cracking resistance will be improved [41].

Figure 5b shows that with the increase in the content of basalt fiber, the cracking area of concrete gradually decreased. When the basalt fiber content reached 0.4%, the cracking area accounted for 56.5% of the concrete with 0.1% basalt fiber content. The elastic modulus of basalt fiber is larger than that of concrete, the interface between basalt fiber and concrete is well bonded, the tensile strength can be improved. Moreover, the crack energy of concrete can be consumed by basalt fiber in the plate test. Therefore, with the increase in the content, the crack area gradually decreases.

As illustrated in Figure 5c, as the content of expander increased, the cracking area of concrete gradually decreased. When the expander content reached 10%, the cracking area accounted for 60.5% of the concrete with 4% expander content. The addition of expander can produce a large number of crystals to fill the pores, and the expansion makes the pressure around the plate offset the shrinkage of concrete. As a result, with the increase in the content of expander, the cracking area of concrete gradually decreased within the scope of this experiment.

Figure 5d shows that as the content of water reducer increased, the cracking area of concrete gradually increased. When the water-reducer content reached 1.3%, the cracking area accounted for 105.7% of the concrete with 1.0% water-reducer content. With the increase in the content of water reducer, the self-shrinkage of the concrete will increase, resulting in the increase in the cracking area of the concrete.

### 3.4. Effect of Material Composition on Microstructure

#### 3.4.1. SEM Analysis

**Figure 6:**
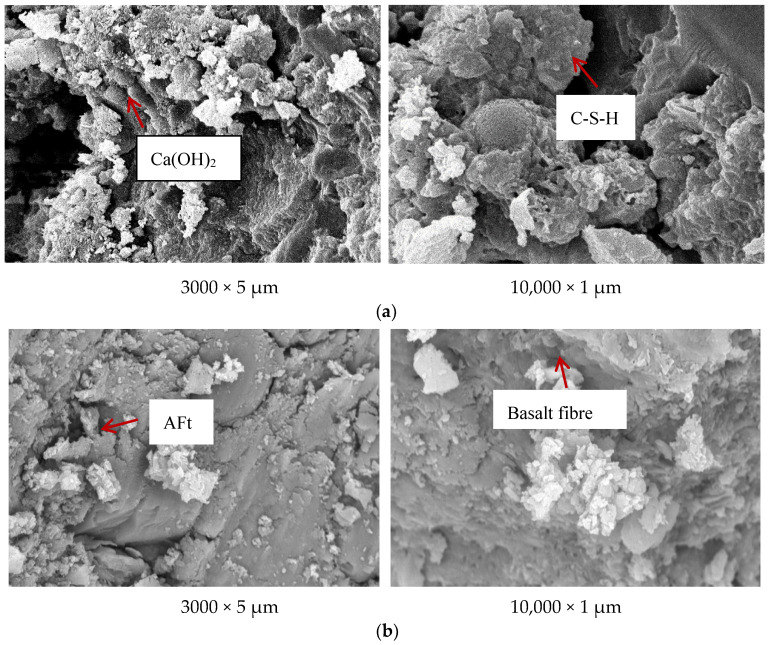
SEM image: (**a**) BG and (**b**) OG.

The microstructure of the HPC was analyzed by scanning electron microscope. BG is the reference concrete, and OG is the optimal mix HPC in Figure 6.

Figure 6 shows that the internal hydration products of BG concrete were mainly C-S-H gels, calcium hydroxide and calcium aluminate hydrate crystals, and the spherical granular fly ash distributed in hydration products. The whole structure was relatively loose with many pores, and the structure was not dense; as can be seen from the SEM of OG concrete, in addition to the same hydration products as BG concrete, there were also substances such as Ettringite (Aft) filled into the pores, and the fact it was interlaced with fibers increased the density of the structure. At the same time, basalt fibers with three-dimensional disorderly distribution could be seen in the image and made the structure more compact.

#### 3.4.2. XRD Analysis

**Figure 7:**
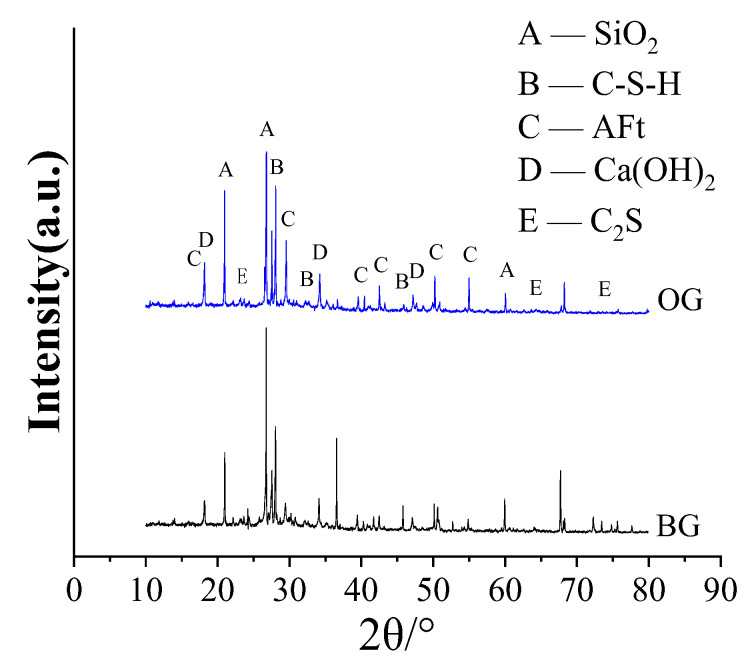
XRD pattern of the HPC.

The X-ray diffraction analysis of the high-performance concrete was performed. BG is the reference concrete, and OG is the optimal mix HPC in Figure 7.

Figure 7 displays the XRD patterns of BG and OG concrete. In the range of 10° < 2θ < 80°, both BG and OG concretes have characteristic peaks of SiO_2_, C-S-H, Aft, Ca(OH)_2,_ and C_2_S. The main components of sand was SiO_2_; C-S-H, AFt, and Ca(OH)_2_ were hydration products; and C_2_S was the clinker of cement. The positions of the characteristic peaks of each phase of the two groups of concrete basically coincided, and no new phase was found. The diffraction peak of C_2_S in OG concrete became lower than BG concrete, which indicated that the C2S in the optimal mix concrete was more completely hydrated and the strength was more developed than in BG concrete; the diffraction peaks of AFt and Ca(OH)_2_ in the optimal mix concrete OG became higher, which showed that the addition of expander produced more hydration products.

#### 3.4.3. MIP Analysis

**Figure 8:**
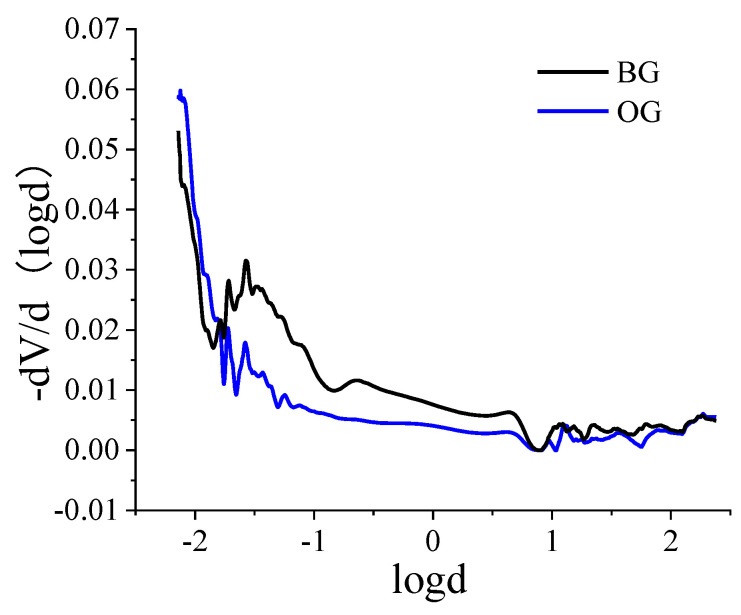
Pore size distribution of the HPC.

In order to analyze the internal pore characteristics of the HPC, mercury injection analysis was carried out. BG is the reference concrete, and OG is the optimal mix HPC. The pore size distribution diagram is shown in Figure 8.

Figure 8 shows that the micro-pores accounted for most of the pore size distribution of BG and OG concrete. Compared with the BG concrete, the OG concrete had more micro-pores and fewer macro-pores, indicating that hydration products such as AFt filled the structural pores and reduced the proportion of macro-pores; as a result, the structure was denser. The basalt fiber also filled the pores, which improved the compactness of the structure, which can improve the strength and crack resistance of the concrete, and the disorderly distributed basalt fiber is also filled in the pores and improves the compactness of the structure, thus improving the strength and crack resistance of the concrete.

## 4. Conclusions

Through the experimental research on the crack resistance of the HPC for the subway underground station floor with different material components, the following conclusions are obtained:

(1) Considering the effect of the fly ash, the basalt fiber, the expander, and the water reducer, the 7 d and 28 d compressive strength of the HPC will gradually decrease with the increase in fly ash and expander content. On the contrary, as the content of basalt fiber increases, the 7 d and 28 d compressive strength of the HPC will gradually increase, but when the content is more than 0.3%, the 28 d compressive strength does not increase significantly. With the increase in the content of the water reducer, the 7 d and 28 d compressive strength of the HPC increases first and then decreases, but the effect on the compressive strength of the HPC is not obvious.

(2) As the content of the fly ash, the basalt fiber, and the expansion agent increases, the initial crack time and through-crack time of the HPC are gradually delayed. On the contrary, with the increase in the content of water reducer, the initial crack time and through-crack time of the concrete will gradually increase.

(3) When the content of the fly ash, the basalt fiber, and the expansive agent increase, the cracking area of the concrete gradually decreases. However, with the increase in the amount of water reducer, the cracking area of the concrete gradually increases. When the water-reducer content reached 1.3%, the cracking area accounted for 105.7% of the concrete with 1.0% water-reducer content.

(4) The addition of the fly ash and the expansive agent can cause a large number of crystalline products to fill the pores, and the cement hydration is more sufficient. At the same time, the disorderly distribution of the basalt fibers can also increase the compactness of the structure; as a result, the proportion of small pores in the concrete increases, and the proportion of large pores decreases significantly, which effectively improves the crack resistance of concrete.

The conclusions obtained can provide guidance for the construction of subway concrete engineering in China and can also be used as a reference for the cracking research of concrete engineering abroad.

## Figures and Tables

**Table 1:** Chemical composition of calcium oxide expander.

Materials	CaO	SiO_2_	Al_2_O_3_	Fe_2_O_3_	L.O.I	∑
Expander	90.14	6.34	0.91	1.24	1.35	99.98

**Table 2:** Mechanical property parameters of basalt fiber.

Length/mm	Filament Diameter/μm	Density/kg·m^−3^	Tensile Strength/MPa	Elastic Modulus/GPa	Elongation /%	Fiberizing Temperature/°C	Max Temperature/°C	Softening Point /°C
12	16	2650	4150	100	3.2	1430	650	960

**Table 3:** Factor level of orthogonal test.

NO.	Fly Ash	Basalt Fiber	Expander	Water Reducer
1	15%	0.1%	4%	1.0%
2	20%	0.2%	6%	1.1%
3	25%	0.3%	8%	1.2%
4	30%	0.4%	10%	1.3%

**Table 4:** HPC mix ratio (kg/m^3^).

NO.	Cement	Fly Ash	Silica Fume	Slag	Sand	Gravel	Water	Basalt Fiber	Expander	Water Reducer
H1	245	75	30	150	646	1054	160	2.65	20	5
H2	245	75	30	150	646	1054	160	5.30	30	5.5
H3	245	75	30	150	646	1054	160	7.95	40	6
H4	245	75	30	150	646	1054	160	10.60	50	6.5
H5	220	100	30	150	646	1054	160	2.65	40	5.5
H6	220	100	30	150	646	1054	160	5.30	50	5
H7	220	100	30	150	646	1054	160	7.95	20	6.5
H8	220	100	30	150	646	1054	160	10.60	30	6
H9	195	125	30	150	646	1054	160	2.65	50	6
H10	195	125	30	150	646	1054	160	5.30	40	6.5
H11	195	125	30	150	646	1054	160	7.95	30	5
H12	195	125	30	150	646	1054	160	10.60	20	5.5
H13	170	150	30	150	646	1054	160	2.65	30	6.5
H14	170	150	30	150	646	1054	160	5.30	20	6
H15	170	150	30	150	646	1054	160	7.95	50	5.5
H16	170	150	30	150	646	1054	160	10.60	40	5
